# Protocol to establish glioma stem cell spheroids and an enriched cancer-associated fibroblast population from a single patient tumor specimen

**DOI:** 10.1016/j.xpro.2026.104359

**Published:** 2026-02-19

**Authors:** Caroline Delmas, Elisabeth Cohen-Jonathan-Moyal, Catherine Seva

**Affiliations:** 1 Centre de Recherche en Cancérologie de Toulouse (CRCT), INSERM U1037, Université de Toulouse, 31000 Toulouse, France; 2 IUCT-Oncopole, Oncopole Claudius Regaud, 31000 Toulouse, France

**Keywords:** Cell culture, Cell isolation, Cancer, Stem Cells

## Abstract

Glioblastoma is an aggressive, therapy-resistant brain tumor in which the role of cancer-associated fibroblasts (CAFs) remains poorly defined. We present a protocol to simultaneously establish glioblastoma stem cell (GSCs) spheroids and CAFs from a single patient specimen. We describe steps to dissociate tumor tissue, generate a single-cell suspension, and establish GSCs spheroids and CAFs cultures. This protocol details CAFs enrichment, passaging, phenotypic characterization, and cryopreservation.

For complete details on the use and execution of this protocol, please refer to Delmas et al.^1^

## Highlights

• Standardized protocol to isolate GSC spheroids and enriched CAFs from single GBM sample
• Steps for tumor dissociation and generation of GSC spheroids and CAF cultures
• Procedure for CAF enrichment, passaging, characterization, and cryopreservation
• Procedure for GSC culture, passaging, characterization, and cryopreservation

## Abstract (editor highlights)

Publisher’s note: Undertaking any experimental protocol requires adherence to local institutional guidelines for laboratory safety and ethics.

## Graphical abstract

**Figure:**
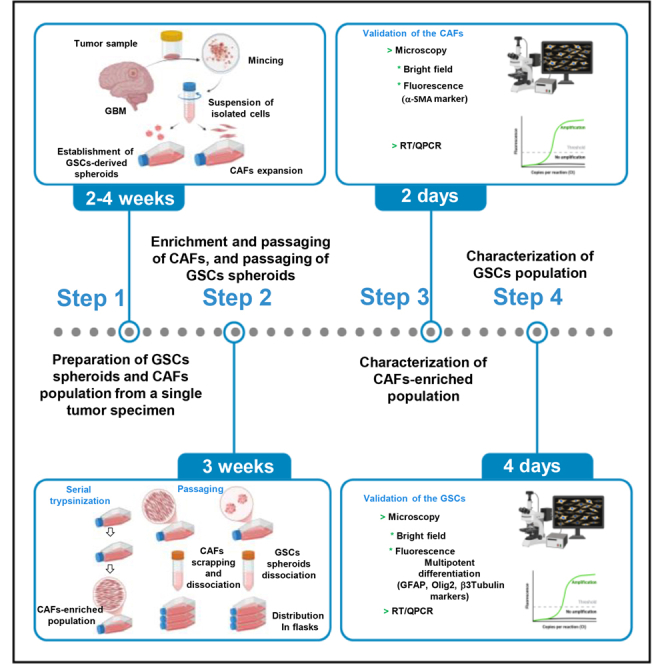

## Teaser

Glioblastoma is an aggressive, therapy-resistant brain tumor in which the role of cancer-associated fibroblasts (CAFs) remains poorly defined. We present a protocol to simultaneously establish glioblastoma stem cell (GSCs) spheroids and CAFs from a single patient specimen. We describe steps to dissociate tumor tissue, generate a single-cell suspension, and establish GSC spheroids and CAF cultures. This protocol details CAF enrichment, passaging, phenotypic characterization, and cryopreservation.

## Before you begin

This protocol outlines methods to establish GSCs spheroids and CAFs from a single patient tumor specimen. It details procedures for enriching and selecting CAFs populations, culturing and passaging both GSCs spheroids and CAFs, and preserving them through cryogenic storage in liquid nitrogen.

All procedures should be performed under sterile conditions in a Class II biological safety cabinet.

### Innovation

Most existing protocols describe either the isolation of GSCs or the isolation of CAFs, but rarely the simultaneous establishment of both populations from a single tumor specimen. This approach enables the study of their interactions within a more patient-relevant context. The co-establishment of GSCs spheroids and CAFs is particularly innovative, as GSCs spheroids retain features of the primary tumor while CAFs represent a major component of the tumor microenvironment. In contrast, the literature predominantly reports separate models or the use of commercial or heterologous CAFs.

Here, we present a protocol that enables the isolation, culture, and maintenance of both GSCs spheroids and CAFs derived from the same patient tumor specimen. The method describes procedures for enriching CAFs populations, establishing and passaging GSCs spheroids, and ensuring long-term storage of both cell types through cryopreservation. By deriving these two complementary populations from a common source, this model provides a valuable platform to study tumor–microenvironment interactions under conditions that more closely recapitulate the patient’s tumor biology. Furthermore, the protocol offers a reproducible and standardized approach that may facilitate translational applications, including drug testing and personalized therapeutics.

### Institutional permissions

This protocol requires tumor tissue that has been surgically resected from patients with GBM. Should you intend to perform experiments utilizing patient specimens, it is essential to secure approval from the appropriate institutions and obtain written informed consent from each individual patient. The GBM patient specimens utilized in this study were gathered from the Department of Neurosurgery at Toulouse University Hospital. The clinical investigators, received authorizations from the Human Research Ethics Committee (Ethics Code: NCT06222138, PI Elizabeth Cohen-Jonathan-Moyal). All participants provided their written informed consent.

### Preparation of culture media

**Timing: 10 min**

1. Preparation of CAFs culture medium.
  a. Prepare DMEM F-12 medium containing 10% FBS by adding 50 mL of FBS to 450 mL of DMEM F-12.

***Note:*** Store at 4°C for up to 1 month.

2. Preparation of GSCs culture medium.
  a. Prepare 40 mL of DMEM F-12 medium containing N2/B27 supplements, FGF-basic and EGF. Detailed recipe is available in the materials and equipment section.

***Note:*** Store at 4°C for up to 1 week.

## Key resources table

**Table:** 

REAGENT or RESOURCE	SOURCE	IDENTIFIER
**Antibodies**		

Anti-αSMA Cy3 (1/400)	Sigma-Aldrich	C6198
Rabbit anti-Olig2 (1/200)	Millipore	AB9610
Rabbit anti-β3Tubulin (1/200)	Abcam	AB18207
Mouse anti-GFAP (1/400)	Chemicon	MAB5628
Donkey anti-Rabbit IgG Alexa Fluor 594 (1/2000)	Invitrogen	A21207
Donkey anti-Mouse IgG Alexa Fluor 488 (1/2000)	Invitrogen	A21202

**Biological samples**		

Human patient materials	Department of Neurosurgery, Toulouse University Hospital	N/A

**Chemicals, peptides, and recombinant proteins**		

DMEM-F12 With L-Gln, 15 mM HEPES, and sodium bicarbonate	Sigma-Aldrich	D8437-500ML
PBS [1X] W CaCl2/MgCl2	Sigma-Aldrich	D8662-500ML
B-27 Serum-Free Supplement (50X), liquid (10 mL)	Thermo Fisher Scientific	17504044
N2 Supplement (100X), liquid (5 mL)	Thermo Fisher Scientific	17502048
FGF-basic	Peprotech	100-18B
EGF	Peprotech	AF-100-15
Penicillin (10000 unit) Streptomycin (10000μg/ml) liquid in 0.9% NaCl	Sigma-Aldrich	P0781-100ML
Fetal Bovine Serum (FBS)	Life-Technology	10270106
Trypsine-EDTA (1X), liquide, 0,05% Trypsine, 0,53 mM EDTA 4Na with phenol red	Sigma-Aldrich	T3924-100ML
DMSO cell culture grade	Sigma-Aldrich	D2650-100ML
BSA	Sigma-Aldrich	
PFA 4%	ChemCruz	Sc281692
Triton X-100	Sigma-Aldrich	X100PC
Prolong Gold Anti-Attenuation Mounting Medium with DAPI	Thermo Fisher Scientific	P36931

**Other**		

Culture dish - 60 x 15 mm	Falcon	353004
T25 Nunc Easy Flasks - 25 cm^2^, with filter cap	NUNC	156367
Cryotube 1.8 ml round bottom with internal screw thread, silicone seal and writing area	NUNC	368632
Conical bottom 15 ml tube	Falcon	352096
Conical bottom 50 ml tube	Falcon	352070
Cell strainer, pore size 100 μm, sterile Corning	MERCK	CLS431752
1.5 mL microtube ClearLine CLEAR-LOCK	Clearline	390901
Cell Scrapers 23 cm Nunc	NUNC	179693
Nunc Lab-Tek Chamber Slide system	Thermo Fisher Scientific	177402
Coverslips 24 x 60 mm	Labelians	LC02460EP1,5n

## Materials and equipment

**Table:** Growth medium for GSCs

Reagent	Final concentration	Amount
DMEM F-12 with L-Gln, Sodium Pyruvate and HEPES	N/A	40 mL
B27 serum free supplement (50X), liquid (10 mL)	1X	0.8 mL
N2 supplement (100X), liquid (5 mL)	1X	0.4 mL
FGF-basic 100 μg/mL	25 ng/mL	0.010 mL
EGF 100 μg/mL	25 ng/mL	0.010 mL

Store at 4°C for up to 1 week.

**Table:** Blocking solution

Reagent	Final concentration	Amount
PBS	N/A	20 mL
BSA	5%	1 g
Triton x100	0.3%	0.060 mL

Store at 4°C for up to 1 week.

## Step-by-step method details

### Preparation of GBM stem cell spheroids and CAFs from a single tumor specimen

**Timing: 1 h 30 min plus 2–4 weeks for spheroid establishment and CAFs expansion**

These steps describe the method for establishing GBM stem cell spheroids and CAFs derived from a single tumor sample of approximatively 100 mm3.

***Note:*** Media volumes and number of culture flasks can be proportionally adapted to smaller or larger tissue samples.

***Note:*** Perform all procedures under sterile conditions and use sterile materials.

1. Obtain sample from surgically resected GBM, collected in 10 mL to 15 mL of PBS.

***Note:*** Samples kept in PBS must be processed within 2 to 4 h. Beyond this time window, and up to 24 h, they may be stored in DMEM-F12 at 4°C.

2. Dissociate tissues for the generation of a single-cell suspension.
  a. Put the tumor specimen with the PBS in a cell culture dish 60 x 15 mm (Figure 1A).

**Figure 1:**
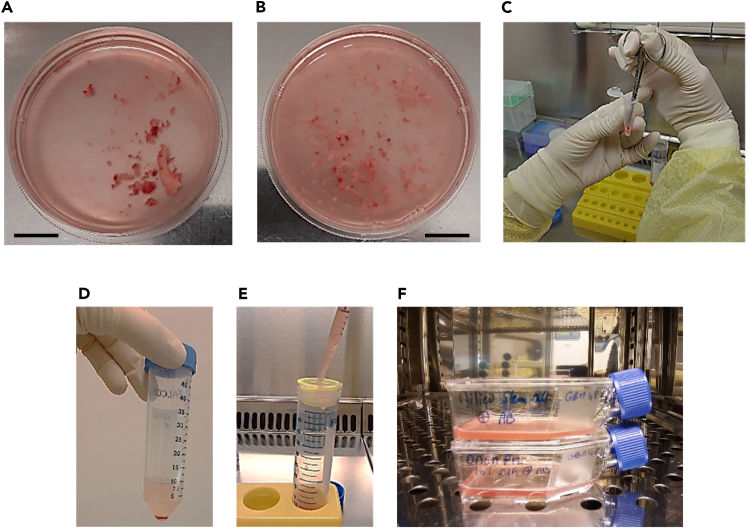
Dissociation of tumor tissues for the generation of a cell suspension Surgically resected GBM sample before mincing (A) and after mincing (B). Scale bar, 1 cm. (C) Difficult-to-cut sections of GBM samples are transferred into an Eppendorf tube and finely minced using sterile scissors. (D) Tumor fragments were agitated 30 minutes in PBS at 37°C then centrifuged. (E) The resulting pellet was resuspended in GSCs growth medium then filtered through a 100 μm cell strainer on a conical 50 mL tube. (F) This cell filtrate is used to prepare 3D culture of GSCs spheroids and the 2D expansion of CAFs.
  b. Cut the tumor specimen into small pieces using sterile scissors (Figure 1B).
    ***Note:*** The pieces must be small enough to pass through the tip of a P1000 pipette.
    ***Note:*** A possible alternative is to use a sterile scalpel instead of scissors to cut the tumor specimen in the cell culture dish.
    ***Note:*** Transfer the pieces that are difficult to cut into a 1.5 mL microtube and cut them with scissors (Figure 1C).
  c. Transfer the tumor fragments with the PBS to a conical 50 mL tube.
  d. Shake the tube at 200 rpm for 30 min. at 37°C.
    ***Note:*** A standard laboratory shaker may be used; no enzymatic digestion is required. There is also no need to use a magnetic stir bar or a GentleMACS dissociator.
  e. Centrifuge the tube at 260 x g for 5 min. at 20°C–25°C.
  f. Remove the supernatant (Figure 1D).
  g. Resuspend the pellet in 6 mL of growth medium for GSCs containing a penicillin-streptomycin mixed solution at 1%.
  h. Filter with a 100 μm sterile cell strainer on a conical 50 mL tube (Figure 1E).
    ***Note:*** This cell filtrate will be used to prepare 3D culture of GSCs spheroids and the 2D expansion of CAFs (Figure 1F).
3. Establish 3D cultures of GSCs-derived spheroids.
  a. Add 5 mL of cell filtrate (from step 2.h.) to a T25 flask for the growth of GSCs spheroids.
    ***Note:*** Keep the remaining 1 mL for 2D CAFs expansion.
  b. Transfer the flask in a 37°C incubator with 5% CO_2_ and 95% humidity for 24h (Figure 1F).
    ***Note:*** After 24 h, most cells in the GSCs medium remain in suspension.
  c. Transfer the cells in suspension to a conical 15mL tube.
  d. Centrifuge the tubes at 260 x g for 5 min. at 20°C–25°C.
  e. Remove the supernatant.
  f. Add 5 mL of fresh growth medium for GSCs containing a penicillin-streptomycin mixed solution at 1%.
  g. Transfer the cell suspension to a new T25 flask.
  h. Transfer the T25 flask to a 37°C incubator with 5% CO_2_ and 95% humidity.
  i. Replace the medium twice a week as described in steps 3c to 3h.
    ***Note:*** When spheroids reach a size of 300-400 μm and their amount increase significantly (2 to 4 weeks), (Figure 2), GSCs can be frozen for storage in liquid Nitrogen. Transfer 4.5 mL of GSCs spheroids suspension in a conical 15 mL tube and add 0.5 mL of dimethyl sulfoxide (DMSO). Mix gently. Prepare 5 cryotubes and add to each tube 1mL of cell suspension. Keep the cryotubes at −80°C for 24 h. Transfer the cryotubes to liquid Nitrogen.
    **Figure 2:**
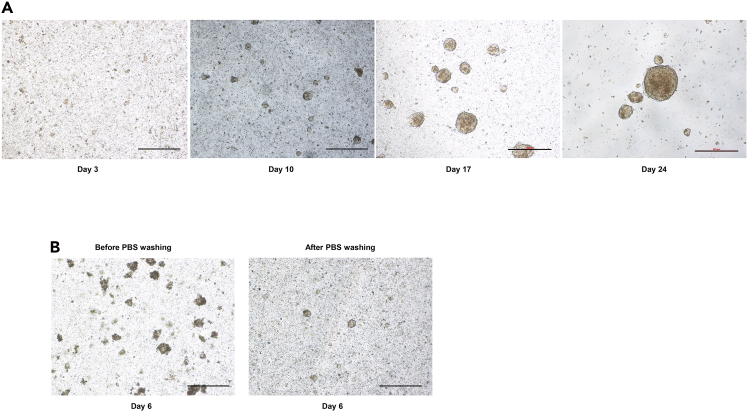
Observation under bright field microscopy of GSCs spheroids (A) Observation under bright field microscopy of GSCs spheroids at days 3, 10, 14, 17 and 24. Scale bar, 500 μm. (B) during the 3D culture of GSCs-derived Spheroids, the medium may initially appear highly turbid due to the presence of suspended cellular debris and numerous red blood cells. This problem can be resolved by including an additional PBS wash.
4. Expand CAFs under two-dimensional culture conditions.
  a. Prepare a T25 flask with 4 mL of CAFs culture medium containing a penicillin-streptomycin mixed solution at 1%.
  b. Add 1 mL of cell filtrate (from step 2.h.) to the flask for the growth of CAFs.
  c. Transfer the flasks to a 37°C incubator with 5% CO_2_ and 95% humidity for 24 hours (Figure 1F).
    ***Note:*** After 24 h, most cells in the CAFs culture medium are adherent to the flask.
  d. Remove the medium and replace with 5 mL of fresh CAFs culture medium.
  e. Change the CAFs culture medium twice a week until the cells reach confluence (2 to 4 weeks).
    ***Note:*** At this stage of the procedure, the adherent cells are heterogeneous including CAFs, tumor glial cells and non-CAFs stromal cells such as macrophages (Figure 3). CAFs population will be enriched in the next step of the protocol.
    **Figure 3:**
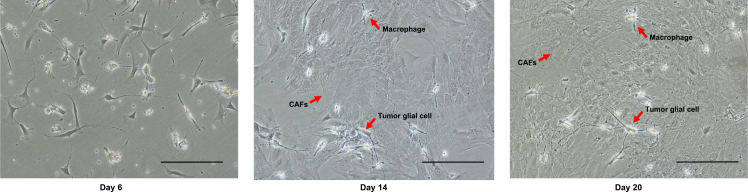
Observation under bright field microscopy of the heterogeneity within the population of adherent cells cultured in CAF medium at days 6, 14, 20 before serial trypsinization Before serial trypsinization, adherent cells in CAFs medium are heterogeneous including CAFs, tumor glial cells and macrophages. Scale bar, 300μm.

### Enrichment and passaging of CAFs and passaging of GSC spheroids

**Timing: 3 weeks for enrichment + 15 min per passage**

These steps describe how to enrich the CAFs population by serial trypsinizations and how to passage the cultures.

5. Perform enrichment of the CAFs population.
  a. Remove the medium from the T25 flask containing the adherent confluent CAFs.
  b. Wash with 2 mL of PBS.
  c. Add 500 μL of trypsin-EDTA (1X) for 5 minutes at 20°C–25°C.
    ***Note:*** The least adherent cells, which included primarily tumor cells and non-CAFs stromal cells such as macrophages, and endothelial cells will detach with this procedure.
  d. Remove the trypsin-EDTA (1X) containing the detached cells by aspiration.
  e. Scrape the highly adherent CAFs with a cell scraper.
  f. Transfer the cells to a 15 mL tube.
  g. Add 10 mL of CAFs medium to obtain a CAFs suspension.
  h. Prepare two T25 flasks and split 5 mL of CAFs suspension per flask.
  i. Transfer the flasks to a 37°C incubator with 5% CO_2_ and 95% humidity.
  j. Change the CAFs medium twice a week.
    ***Note:*** From this step, remove penicillin-streptomycin mixed solution from the CAFs culture medium.
    **CRITICAL:** Repeat this process of trypsinization following confluence, along with the scraping of the CAFs (steps 5.a to 5.i) at least three times to achieve a uniform population resistant to trypsinization and exhibiting a fibroblastic morphology (3 weeks) (Figure 4A).
    **Figure 4:**
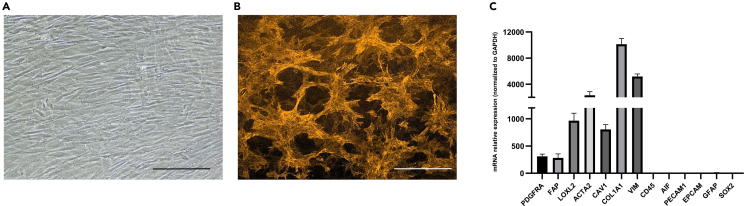
Characterization of the enriched CAFs population (A) Typical morphology of CAFs under bright field microscopy. Scale bar, 300 μm. (B) Immunofluorescence staining of the enriched CAFs population with a CAFs marker, αSMA. Scale bar, 300 μm. (C) Enriched-CAFs populations were analyzed by real time PCR for the expression of established CAFs markers such as ACTA2, FAP, LOXL2, COL1A1, PDGFRA, CAV1 and VIM, while also ensuring the absence of markers associated with non-CAFs stromal cells that exhibit similarities with CAFs. These include markers for epithelial cells (EPCAM), endothelial cells/pericytes (PECAM1), and immune cells (CD45, AIF1) as well as GBM cells/stem cells (GFAP, Sox2). Relative expressions on 17 different patients are presented as means ± SD.
6. Perform passaging of GSC spheroids.
  ***Note:*** When spheroids attain a diameter of approximately 300-400 μm, or when the core of the spheroid exhibits a dark coloration (signifying the presence of necrotic cells), and when their quantity increases substantially, it is necessary to passage the spheroids.
  a. Transfer the 5 mL of spheroids suspension to a conical 15 mL tube.
  b. Centrifuge the spheroids suspension at 260 x g for 5 min. at 20°C–25°C.
  c. Remove the supernatant.
  d. Add 200 μL of trypsin-EDTA (1X).
  e. Disrupt spheroids by performing up and down pipetting 20 times with a p200 pipette.
  f. Incubate for 2 min. at 20°C–25°C.
  g. Add 800 μL of PBS.
  h. Disrupt spheroids by performing up and down pipetting 20 times with a p200 pipette.
  i. Centrifuge the suspension at 260 x g for 5 min. at 20°C–25°C.
  j. Remove the supernatant.
  k. Resuspend the pellet in 10 mL of growth medium for GSCs without penicillin-streptomycin mixed solution.
  l. Split 5 mL/flask in 2 separate T25 flasks.
  m. Change the GSCs medium twice a week.
    ***Note:*** From the first passage, remove the penicillin-streptomycin mixed solution from the GSCs culture medium.
7. Perform passaging of the enriched CAFs culture.
  a. Remove the medium from the T25 flask containing the adherent confluent CAFs.
  b. Wash with 2 mL of PBS.
  c. Add 500 μL of 0.25% trypsin-EDTA for 5 min. at 20°C–25°C.
  d. Remove the trypsin by aspiration.
  e. Scrape the highly adherent CAFs with a cell scraper.
  f. Transfer to a 15 mL tube.
  g. Add 10 mL of CAFs growth medium to obtain a CAFs suspension.
  h. Split 5 mL/flask in 2 separate T25 flasks.
  i. Transfer the flasks in a 37°C incubator with 5% CO_2_ and 95% humidity.
  j. Change the CAFs growth medium twice a week.

### Characterization of the enriched CAFs population

**Timing: 2 days**

These steps describe how to characterize the enriched CAFs population.

8. Observe the cells using bright-field microscopy.

***Note:*** To validate the enrichment in CAFs, verify the typical morphology of CAFs under bright field microscopy (Figure 4A).

***Note:*** Any microscope that has brightfield functions can be used.

9. Perform Immunostaining.
  a. When the enriched CAFs culture reaches 80% confluence, proceed with the steps described above (7a–7g).
  b. Count the cells using a standard cell counter.
  c. Adjust the CAFs suspension to a concentration of 10.000 cells/mL.
  d. Seed 500 μL of the cell suspension into a chamber of a Lab-Tek chamber slide.
  e. When the culture reaches 80% confluence, remove the culture medium.
    i. Fix adherent cells by adding 100 μL of 4% PFA solution.
    ii. Incubate for 15 min. at 20°C–25°C.
  f. During the 15 min. of incubation, prepare 20 mL of blocking solution (Detailed recipe is available in the materials and equipment section).
  g. Remove the PFA 4% solution after the 15 min. of incubation.
    i. Wash twice with 500 μL PBS for 5min.
    ii. Add 500 μL of blocking solution.
    iii. Incubate for 1hour at 20°C–25°C.
  h. Remove the blocking solution.
    i. Add 100 μL of anti-αSMA–Cy3 antibody diluted at 1/400 in blocking solution.
    ii. Incubate for 18 hours at 4°C, protected from light.
  i. Remove the antibody solution.
  j. Wash twice with 500 μL PBS for 5 min.
  k. Mount coverslips using ProLong™ Gold anti-attenuation mounting medium with dapi.
  l. Allow slides to cure overnight before fluorescence microscopy observation (Figure 4B).

***Note:*** Any microscope that has fluorescence functions can be used.

***Note:*** You may optimize the use of your 8-wells Lab-Tek chamber by seeding the remaining wells in the same manner if you wish to perform additional immunostainings on these cells or if you have CAFs derived from multiple patient samples to characterize.

***Note:*** We recommend conducting RT/QPCR on the CAFs-enriched cell population to verify the elevated expression of established CAFs markers such as ACTA2, FAP, LOXL2, COL1A1, PDGFRA, CAV1 and VIM, while also ensuring the absence of markers associated with non-CAFs stromal cells that exhibit similarities with CAFs. These include markers for epithelial cells (EPCAM), endothelial cells/pericytes (PECAM1), and immune cells (CD45, AIF1) as well as GBM cells/stem cells (GFAP, Sox2) (Figure 4C).

***Note:*** Any standard RT-qPCR protocol can be used to assess the expression of the markers listed above.

***Note:*** After their Characterization, CAFs can be frozen for storage in liquid Nitrogen. Remove the culture medium from the T25 flask. Wash with 2 mL of PBS. Add 500 μL of 0.25% trypsin-EDTA for 5 min at room temperature. Remove the trypsin. Scrape the adherent CAFs with a cell scraper and transfer into a 15mL tube. Add 4.5mL of CAFs medium with 0.5mL of dimethyl sulfoxide (DMSO). Mix gently. Transfer to 5 cryotubes 1mL of cell suspension. Keep the cryotubes at −80°C for 24 h. Transfer the cryotubes to liquid Nitrogen.

### Characterization of the GSCs population

**Timing: 4 days**

These steps describe how to characterize the GSCs population by their capacity to generate spheroids under stem cell culture conditions, to express stem cell markers, and to undergo multipotent differentiation into the neuronal, oligodendroglial, and astrocytic lineages.

10. Bright field microscopy.

**Figure 5:**
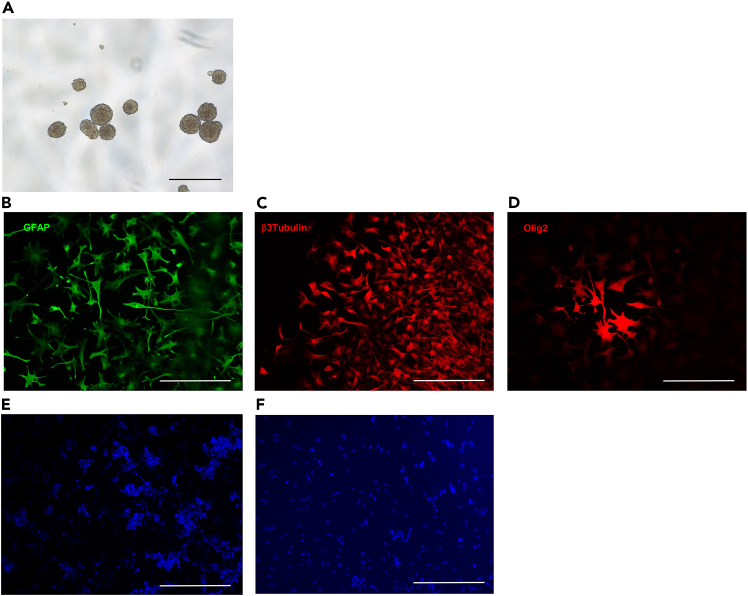
Characterization of the GSCs population (A) Typical morphology of GSCs spheroids under bright field microscopy. Scale bar, 500 μm. (B-F) GSCs differentiated in FBS. (B–D) Immunofluorescence staining shows multipotent differentiation into neuronal (β3Tubulin marker), oligodendroglial (Olig2 marker), and astrocytic (GFAP marker) lineages. (E) Negative control image obtained using donkey anti-rabbit IgG Alexa Fluor® 594 in the absence of primary antibody. (F) Negative control image obtained using donkey anti-mouse IgG Alexa Fluor® 488 in the absence of primary antibody. Scale bar, 300 μm.

To validate the GSCs phenotype, verify their capacity to generate spheroids under stem cell culture conditions under bright field microscopy (Figure 5A).

11. Immunofluorescence on differentiated GSCs population.
  a. After completing steps 6a through 6j of the passaging procedure for GSCs, resuspend the pellet in 10 mL of DMEM-F12 containing 10% FBS.
  b. Count the cells using a standard cell counter.
    i. Adjust the GSCs suspension to a concentration of 20,000 cells/mL.
    ii. Seed 500 μL of the cell suspension into 5 chambers of a Lab-Tek chamber slide.
  c. Maintain the cells in this medium in a 37°C incubator with 5% CO_2_ and 95% humidity for 96 hours.
  d. Remove the culture medium.
    i. Fix adherent cells by adding 100 μL of 4% PFA solution.
    ii. Incubate for 15 minutes at 20°C–25°C.
  e. Prepare 20 mL of blocking solution (Detailed recipe is available in the materials and equipment section).
  f. Remove the PFA 4% solution.
    i. Wash twice with 500 μL PBS for 5 min.
    ii. Add 500 μL of blocking solution.
    iii. Incubate for 1 hour at room 20°C–25°C.
  g. Remove the blocking solution.
    i. Add in 3 chambers 100 μL of primary antibody diluted in blocking solution: anti-GFAP (1/400), anti-β3 Tubulin (1/200), or anti-Olig2 antibody (1/200).
    ii. Add 100 μL of blocking solution to the 2 remaining chambers.
      ***Note:*** These two chambers serve as negative controls and do not receive primary antibodies.
  h. Incubate overnight at 4°C, protected from light.
  i. Remove the solution in each chamber.
    i. Wash twice with 500 μL PBS for 5 min.
  j. Prepare the appropriate fluorochrome-conjugated secondary antibodies in blocking buffer (1/2000).
    i. Add 200 μL per chamber.
    ii. Incubate for 1 hour, at 20°C–25°C, in the dark.
  k. Remove the secondary antibody solution.
    i. Wash twice with 500 μL of PBS for 5 min.
  l. Mount coverslips using ProLong™ Gold anti-attenuation mounting medium with dapi.
  m. Allow slides to cure 18 hours before fluorescence microscopy observation (Figures 5B–5F).
    ***Note:*** Any microscope that has fluorescence functions can be used.
    ***Note:*** You may optimize the use of your 8-wells Lab-Tek chamber by seeding the remaining wells in the same manner if you wish to perform additional immunostainings on these cells or if you have GSCs derived from multiple patient samples to characterize.
    ***Note:*** RT/QPCR or western blots can be conducted on the GSCs population to verify the expression of established GSCs markers such as SOX2, Nestin, ITGA6.^2^ Any standard RT-qPCR or western blot protocol can be used to assess the expression of the markers listed above.
    ***Note:*** Extreme Limiting Dilution Analysis (ELDA) can be performed to assess GSCs self-renewal potential,^3^ following a previously published step-by-step protocol.^4^

## Expected outcomes

Expected outcomes include obtaining an enriched population of CAFs derived from GBM patient samples, exhibiting characteristic CAFs morphology, robust expression of established CAFs markers, and the absence of markers associated with non-CAFs stromal cell populations, consistent with the findings of Delmas C et al.^1^ In parallel, the generation of glioblastoma stem cells (GSCs) spheroids suitable for resistance testing to treatments, including radiotherapy as well as invasion assays when embedded in Matrigel, is expected.^5^^,^^6^

Importantly, obtaining GSCs and CAFs spheroids from the same glioblastoma patient provides an experimental model that more accurately replicates the patient’s tumor biology. This approach enables consideration of a part of the tumor microenvironment and strengthens the translational relevance of downstream applications, including drug screening and personalized therapeutic strategies.

## Limitations

Despite its ability to generate patient-derived GSCs spheroids and cancer-associated fibroblasts (CAFs) from a single tumor specimen, this protocol presents several limitations. First, it requires access to freshly resected tumor tissue of sufficient volume and viability, which can limit reproducibility across samples and institutions. The establishment and expansion of both GSCs spheroids and CAFs are time-consuming, typically taking several weeks, and may fail depending on the intrinsic cellular composition or quality of the original specimen.

## Troubleshooting

### Problem 1

Tumor specimens collected from patients may occasionally be excessively large, which can hinder the efficient preparation of both spheroids and CAFs (related to step 1).

### Potential solution

For optimal generation of GBM stem cell spheroids and CAFs from a single tumor sample, we recommend to process tissue fragments not exceeding approximately 500 mm^3^ in volume.

### Problem 2

During step 2 of the protocol, the GSCs culture medium may initially appear highly turbid due to the presence of suspended cellular debris and numerous red blood cells. This condition may persist for one to two weeks, until the spheroids progressively enlarge and become the predominant structures in the culture (Figure 2B).

### Potential solution

After step 2d, include an additional PBS wash by adding 5 mL of PBS, followed by centrifugation for 5 minutes. Carefully remove the PBS and proceed with step 2e (Figure 2B). Repeat this washing procedure for one to two weeks as needed.

### Problem 3

During step 3, the number of adherent cells may remain relatively low during the initial two weeks of culture.

### Potential solution

Wait approximately four weeks for a marked exponential increase in cell growth.

### Problem 4

At step 3d, culture medium may appear highly turbid due to the presence of numerous red blood cells.

### Potential solution

Wash the adherent cells with 5 mL of PBS before adding 5 mL of fresh CAFs culture medium.

### Problem 5

With increasing passages, CAFs and GSCs cultures may undergo phenotypic drift.

### Potential solution

To minimize this issue, limit the number of passages to ten or fewer.

## Resource availability

### Lead contact

Further information and requests for resources and reagents should be directed to and will be fulfilled by the lead contact, Catherine Seva (cathy.seva@inserm.fr).

### Technical contact

Technical questions on executing this protocol should be directed to and will be answered by the technical contact, Caroline Delmas (delmas.caroline@iuct-oncopole.fr).

### Materials availability

This study did not generate new or unique reagents.

### Data and code availability

This study did not generate a dataset or code.

## Acknowledgments

This work was supported by INSERM (“Institut National de la Sante et de la Recherche Medicale”) and La Ligue contre le Cancer LNCC31 (2025).

## Author contributions

C.S. drafted and wrote the manuscript. C.D. performed the experiments. E.C.-J.-M. conducted the clinical investigation (NCT06222138). C.D., E.C.-J.-M., and C.S. reviewed and proofread the manuscript.

## Declaration of interests

The authors declare no competing interests.
